# Is there a causal nexus between COVID-19 infection, COVID-19 vaccination, and Guillain-Barré syndrome?

**DOI:** 10.1186/s40001-023-01055-0

**Published:** 2023-02-25

**Authors:** Xiaoxiao Zheng, Yong Fang, Yanna Song, Shan Liu, Kangding Liu, Jie Zhu, Xiujuan Wu

**Affiliations:** 1 Department of Neurology, Neuroscience Center, The First Hospital of Jilin University, Jilin University, Xinmin Street 1#, Changchun, 130021 China; 2 Department of Neurology, The Third Affiliated Hospital of Sun Yat-sen University, Guangzhou, China; 3 The Second Hospital of Jilin University, Jilin University, Changchun, China; 4 Department of Neurobiology, Care Sciences and Society, Karolinska Institute, Karolinska University Hospital, Solna, Stockholm, Sweden

**Keywords:** Guillain-Barré syndrome, SARS-CoV-2, COVID-19, Vaccination, Epidemiology

## Abstract

Guillain-Barré syndrome (GBS) is an immune-mediated inflammatory polyradiculoneuropathy, which commonly leads to a very high level of neurological disability. Especially, after the global outbreak of severe acute respiratory syndrome coronavirus 2 (SARS-CoV-2) infection, the causation between GBS and SARS-CoV-2 infection and the coronavirus disease 2019 (COVID-19) vaccination have aroused widespread concern. In the review, we analyzed the impacts of SARS-CoV-2 infection and COVID-19 vaccination on GBS globally, aiming to further understand the characteristics of GBS associated with COVID-19. Based on the electrophysiological data, patients suffering from GBS related to COVID-19 manifested as an acute inflammatory demyelinating polyneuropathy (AIDP). Moreover, we summarized the current findings, which may evidence GBS linking to SARS-CoV-2 infection and COVID-19 vaccination, and discussed the underlying mechanisms whether and how the SARS-CoV-2 virus and COVID-19 vaccination can induce GBS and its variants.

## Introduction

Guillain-Barré syndrome (GBS) is an acute inflammatory peripheral radiculopathy and neuropathy characterized by progressive limb weakness, sensory deficits, cranial nerve involvement, tendon areflexia, and cerebrospinal fluid (CSF) albuminocytological dissociation with a reported global incidence of approximately 100,000 new cases annually [1]. It is reported that up to 70% of patients developed preceding infections in the upper respiratory and digestive tracts within 2 weeks before the onset of neurological signs and symptoms. *Campylobacter* *jejuni (C. jejuni)* is the most common pathogens causing the antecedent infection of GBS, which is thought to be induced through molecular mimicry between the lipopolysaccharides (LPSs) of *C.* *jejuni* and the gangliosides of the nerve system [2]. In other words, *C.* *jejuni* LPSs result in the production of antibodies response to nerve gangliosides by molecular mimicry. According to the pathology, clinical manifestations, and neurophysiological characteristics, GBS is mainly divided into several variants, which mainly includes acute inflammatory demyelinating polyneuropathy (AIDP), acute motor axonal neuropathy (AMAN), and acute motor and sensory axonal neuropathy (AMSAN) [3]. Multidisciplinary medical care and immunotherapy, such as intravenous immunoglobulin (IVIG) and plasma exchange (PE), are optimal treatments in GBS [2]. Most GBS patients get a full recovery, while some patients developed chronic disabilities and 3–10% of cases die during the acute phase [4]. The novel outbreak caused by severe acute respiratory syndrome coronavirus 2 (SARS-CoV-2) infection, named as coronavirus disease 2019 (COVID-19), usually manifests with respiratory symptoms, and neurological harm, such as GBS. With the global wide vaccination of COVID-19 vaccine, GBS cases after vaccination have gradually increased. Although extensive attention has been attracted to COVID-19 and its vaccine-associated GBS, there was still lack of the pathogenic mechanism between GBS and SARS-CoV-2 infection or COVID-19 vaccination. In this review, we summarized the clinical characteristics of GBS after SARS-CoV-2 infections and COVID-19 vaccination and discussed the updated knowledge about the possible relationship between GBS and SARS-CoV-2 infection or COVID-19 vaccination, as well as the behind pathogenic mechanisms, aiming to further deepen the understanding of COVID-19-associated GBS and provide potential precise treatment worldwide.

## Epidemiology

GBS is a common cause of acute flaccid paralysis with a crude incidence of 0.16 to 3.0 cases per 100,000 person-years worldwide. The annual global incidence of GBS, based on North American and European studies, mainly fluctuates from 0.81 to 1.91 cases per 100,000 person-years in adults and about 0.6 per 100,000 person-years in children [1]. The variation in incidence may be partly associated with the geographical area because of differences in genetic susceptibility or exposure to pathogenic bacteria among different populations [5].

GBS can occur at any age. Many epidemiological studies of GBS revealed that incidence of GBS increased with age after 50 years, increasing by 20% for each additional decade of age [1].

In Italy, the monthly incidence during March–April period of 2017–2019 years was 0.12 new cases/100,000 inhabitants per month, and the increase of GBS cases in 2020 is 5.41-fold [6]. Another study in northern Italy also suggested an increased incidence of GBS from March to April 2020 compared with that in the same months in 2019 (rate per year estimated to be 2.43/ 1,000,000 and 0.93/1,000,000 in 2020 and 2019, respectively) [7]. Fragiel and his colleagues identified 11 cases of GBS among 71,904 COVID patients who were treated in the Spanish emergency departments (EDs) during the 2-month pandemic peak. The relative frequency of GBS in COVID and non-COVID was 0.15 versus 0.02 among EDs patients, as was the standardized incidence (9.44cases/100,000 inhabitant-years versus 0.69 cases/100,000) [8]. As mentioned above, the overall number of GBS admissions has been reduced during the COVID outbreak. In addition to wash hands frequently, pay attention to hygiene and use preventive measures to minimize the risk of infection, potential reasons include the mildest cases did not seek medical attention but remained at home, due to the fear of contracting the coronavirus.

## GBS and COVID-19

SARS-CoV-2 is a member of the betacoronavirus genus, which are enveloped viruses with a positive-sense ribonucleic acid (RNA)-strand genome [9]. The COVID-19 caused by SARS-CoV-2 has led to a global pandemic outbreak in the twenty-first century. Except for severe respiratory symptoms, COVID-19 could trigger neurological harm, such as myositis, myasthenia gravis, stroke, encephalitis, acute meningitis, and also GBS [10, 11]. Over 200 cases of GBS following COVID-19 infection have been reported from Europe, America, India, Brazil, Iran, China, and the Middle East. So far, at least 23 countries reported GBS associated with COVID-19: 80 cases in Italy [6, 7, 12], 41 cases in the United Kingdom (UK) [13–15], 25 cases in Spain [8, 14, 16, 17], 30 cases in the United States (USA) [15, 16, 18], 16 cases in Iran [16], 12 cases in India [14–16], 6 cases in France, 4 cases in Belgium, Germany, and Switzerland, respectively, 2 cases in China, and Guinea, Austria, Brazil, Canada, Columbia, Japan, Morocco, Netherlands, Sudan, Tanzania, Turkey, and Saudi Arabia reported one case, respectively [16].

A meta-analysis in 2021 revealed that the overall prevalence of GBS among the COVID-19 patients were 15/100,000 population-years [14]. In January 2020, the first case of GBS after COVID-19 infection was reported in China, mainly with acute weakness in both legs, distal hypoesthesia, like typical demyelinating characteristics, and albumin-cytologic dissociation of CSF, which alerted clinicians to the potential link between COVID-19 and GBS [19]. Considering the onset of GBS symptoms in this patient overlapped with the period of COVID-19 infection, it suggested that COVID-19-GBS associated with COVID-19 might follow parainfective mechanism. Currently, the correlation between COVID-19 infection and GBS remains controversial.

GBS is featured by rapidly progressive flaccid paralysis of the limb with areflexia or hyporeflexia. The onset of GBS is acute or subacute, and the disease reaches its nadir within 2–4 weeks [20]. In typical GBS, sensory disturbance, including pain and numbness, and muscle weakness in limbs are usually the first symptoms. Rapidly progressive weakness, the most common clinical characteristic of GBS, mainly affects the distal or proximal muscles of limbs, accompanied by distal paresthesia or sensory deficits. More than 50% of GBS patients have cranial nerve deficits, mainly involving bilateral facial nerves, oculomotor nerves, glossopharyngeal nerves, and vagus nerves, which resulted in bilateral facial weakness, bulbar palsy, and ophthalmoplegia, respectively [21]. Currently available 204 cases of GBS following COVID-19 infection are summarized in Table 1 with a comparison of clinical characteristics between different countries (Table 1). The median age of patients was 57 years, and males are at higher risk than women, with a male-to-female ratio of 2.4 to 1. Most patients developed neurological symptoms within 2 weeks after COVID-19 infection, while patients in India tend to present with neurological symptoms about 1 week after COVID-19 infection. The clinical manifestations, electrophysiological findings, and therapies of COVID-19-related GBS resembled those of International Guillain-Barre´ syndrome Outcome Study (IGOS), with limb weakness as the most typical symptom, AIDP as the most common subtype, and IVIG as the main treatment. However, the frequency of autonomic dysfunction among patients with GBS after COVID-19 infection varies considerably between regions, with a higher proportion in America (48%) and a lower incidence in Spain (7%). Several rare variants, including Miller Fisher syndrome (MFS), pure sensory GBS, pharyngeal-cervical-brachial (PCB) variant, acute panautonomic neuropathy, pharyngeal-cervical-brachial weakness, and paraparetic GBS, have been reported in GBS following COVID-19 infection [6, 7, 22–24]. Besides, axonal GBS (AMAN/AMSAN) are more frequent in Iran and India, and their proportion is comparable to that of AIDP in their regions. Compared with the results of IGOS, more than 30% of patients required mechanical ventilation. In addition to respiratory muscle weakness caused by GBS, the high proportion of ventilator dependency may also be attributed to acute lung injury caused by COVID-19. Besides, the prognosis of COVID-19-triggered GBS is worse than that of non-COVID-19 GBS, which may be related to systemic damage caused by COVID-19. It is important to interpret the results with caution due to additional studies with a larger sample size are needed to verify these results [25].

**Table 1:** Clinical features of GBS following COVID-19 infection in different countries

GBS features	All (*n* = 204)	Italy (*n* = 80)	UK (*n* = 41)	USA (*n* = 30)	Spain (*n* = 25)	Iran (*n* = 16)	India (*n* = 12)
Demographics							
Age (years), median (IQR)	57 (48–65)	62 (55–72)	57 (43–60.75)	55 (48–67)	54.5 (51.75–64.75)	46.5 (37.75–65)	48.5 (33–55)
Male: female ratio	144/60 (2.4)	57/23 (2.5)	36/5 (7.2)	20/10 (2)	14/11 (1.3)	9/7 (1.3)	8/4 (2)
Clinical features							
The interval from onset of COVID symptoms to neurological symptoms (days), median (IQR)	13.5 (7–20)	13.5 (10–20)	13 (6.5–20.25)	17.5 (9.5–41.5)	11 (6.25–15.75)	14 (9.5–21)	7.5 (5–10)
Limb weakness (%)	166/192 (86)	66/80 (83)	38/41 (93)	26/30 (87)	8/13 (62)	14/16 (88)	12/12 (100)
Sensory disturbances (%)	87/143 (61)	15/33 (46)	29/41 (71)	19/30 (63)	8/14 (57)	11/16 (69)	5/12 (42)
Cranial nerve involvement (%)	102/185 (55)	51/80 (64)	19/41 (46)	12/30 (40)	9/14(64)	7/16(44)	4/12(33)
Ophthalmoplegia	18/158 (11)	8/80 (10)	2/15 (13)	6/30 (20)	2/14 (14)	0/16 (0)	0/12 (0)
Facial weakness	67/159 (42)	36/80 (45)	6/15 (40)	8/30 (27)	7/14 (50)	6/16 (37.5)	3/12 (25)
Bulbar weakness	45/161 (28)	17/80 (21)	8/15 (53)	8/30 (27)	6/14 (43)	4/16 (25)	3/12 (25)
Other	30/161 (19)	19/80 (24)	2/15 (13)	6/30 (20)	2/14 (14)	0/16 (0)	1/12 (8)
Autonomic dysfunction (%)	35/137 (25)	12/63 (19)	6/16 (38)	10/30 (33)	1/14 (7)	5/16 (31)	3/12 (25)
Areflexia or Hyporeflexia (%)	142/148 (96)	60/63 (95)	15/16 (94)	27/30 (90)	13/14 (93)	16/16 (100)	11/12 (92)
Ventilator dependency (%)	56/170 (33)	21/63 (33)	13/41 (32)	10/30 (33)	4/14 (29)	6/16 (37.5)	2/12 (17)
GBS subtypes (%)							
AIDP	91/150 (61)	51/78 (65)	17/21 (81)	5/16 (31)	6/12 (50)	7/12 (58)	5/11 (45)
AMAN	16/150 (11)	8/78 (10)	1/21 (5)	1/16 (6)	0/12 (0)	3/12 (25)	3/11 (27)
AMSAN	13/150 (9)	4/78 (5)	1/21 (5)	3/16 (19)	1/12 (8)	2/12 (17)	2/11 (18)
Miller Fisher syndrome	19/150 (13)	8/78 (10)	2/21 (9)	6/16 (38)	3/12 (25)	0/12 (0)	0/11 (0)
Other	11/150 (7)	7/78 (9)	0/21 (0)	1/16 (6)	2/12 (17)	0/12 (0)	1/11 (9)
Nerve conduction studies (%)							
Demyelination	95/157 (61)	54/76 (71)	17/36 (47)	6/10 (60)	7/10 (70)	6/13 (46)	5/12 (42)
Axonal damage	32/157 (20)	14/76 (18)	3/36 (8)	4/10 (40)	1/10 (10)	5/13 (38)	5/12 (42)
Mixed demyelination and axonal damage	30/157 (19)	8/76 (10)	16/36 (44)	0/10 (0)	2/10 (20)	2/13 (15)	2/12 (17)
Laboratory tests (%)							
CSF albumin-cytological dissociation	93/134 (70)	27/51 (53)	11/14 (79)	20/24 (83)	15/23 (65)	10/11 (91)	10/11 (91)
Anti-ganglioside antibodies in serum or CSF	8/65 (12)	2/28 (7)	0/4 (0)	4/15 (27)	2/10 (20)	0/1 (0)	0/7 (0)
Treatments (%)							
IVIG	155/175 (89)	70/75 (93)	32/32 (100)	22/30 (73)	11/12 (92)	10/15 (67)	10/11 (91)
Plasma exchange	24/175 (14)	7/75 (9)	1/32 (3)	11/30 (37)	0/12 (0)	4/15 (27)	1/11 (9)
Corticosteroids	9/175 (5)	4/75 (5)	0/32 (0)	3/30 (10)	1/12 (8)	1/15 (7)	0/11 (0)
Outcomes (%)							
Recovery^^^	150/193 (78)	63/80 (79)	32/41 (78)	24/30 (80)	10/14 (71)	11/16 (69)	10/12 (83)
Residual problems^#^	33/193 (17)	15/80 (19)	8/41 (20)	4/30 (13)	3/14 (21)	2/16 (12.5)	1/12 (8)
Mortality	10/193 (5)	2/80 (2.5)	1/41 (2.4)	2/27 (7)	1/14 (7)	3/16 (19)	1/12 (8)

^ able to walk independently or symptoms improvement; # unable to walk independently, disability, activity limitations, ventilator dependency, or severe persistent pain and fatigue; Other cranial nerve involvement: facial paresthesia or hypoglossal nerve palsy

Other GBS subtypes: acute sensory neuropathy, acute panautonomic neuropathy, pharyngeal-cervical-brachial weakness, paraparetic GBS, or other variants

*GBS* Guillain-Barré syndrome, *COVID-19* coronavirus disease 2019, *UK* United Kingdom, *USA* the United States of America, *AIDP* acute inflammatory demyelinating polyradiculopathy, *AMAN*, acute motor axonal neuropathy, *AMSAN* acute motor and sensory axonal neuropathy, *CSF* Cerebrospinal Fluid, *IVIG* Intravenous immunoglobulin

### Is there a causal nexus between GBS and COVID-19 infection?

In a retrospective case series by Gigli and others, the authors reported an unusual cluster of seven patients affected by GBS in an Italian region, which coincided with the descending curve of the COVID-19 pandemic [6]. Incidence of GBS in March and April of previous years was 0.12 cases/100,000 versus 0.65 cases/100,000 during the ongoing pandemic and a 5.41-fold increase of GBS cases was noticed in 2020. Furthermore, a study by Tatu and others found that SARS-CoV-2 infection may act as a hypothetical downstream precipitating factor, triggering GBS and other autoimmune diseases [26]. In another retrospective study by Filosto and others showed that a 2.6-fold increase in the incidence of GBS in March–April 2020 compared to March–April 2019 in northern Italy suggesting a pathogenic link and COVID-19-related GBS to be more severe compared to non-COVID-related GBS. COVID-19-positive patients with GBS were characterized by severe clinical pictures affecting mainly the respiratory system, while the mentioned features most likely contributed to the development of COVID-19-driven systemic impairment [7]. Given that the onset of GBS symptoms in the first reported patient with GBS associated with SARS-CoV-2 infection overlapped with the period of SARS-CoV-2 infection, GBS associated with SARS-CoV-2 might follow parainfective mechanism which differs from the classic GBS associated with Zika virus infection [19]. However, the incubation period of COVID-19 is up to 2 weeks making the interval of days between the onset of COVID-19 and the GBS symptoms seem short. In the great majority of patients, the median interval was 13.5 days. Besides, polymerase chain reaction (PCR) for SARS- CoV-2 was negative in CSF in all tested cases. Overall, these findings support the likelihood that postinfective mechanism could play a role than a parainfective. Generally, the case reports did not suggest a proven causal relationship, and further data and analyses will be necessary to precisely determine the GBS and its causal link with SARS-CoV-2.

However, there was no evidence of an association between SARS-COV-2 infection and the development of immune-mediated neuropathies, despite there were reports of GBS and Miller Fisher syndrome during COVID-19 [27]. During the COVID-19 pandemic, the incidence of GBS may have decreased as a result of a possible decline in the transmission of infectious diseases due to COVID-19 prevention measure. A study of patients with GBS in UK hospitals indicated that the number of patients with GBS decreased between March and May 2020, compared with that in the same months in 2016–2019 (1.65–1.88 per 100,000 individuals per year), possibly because reduced social contacts and increased hand hygiene had decreased the circulation of other etiologic agents, such as *C. jejuni* and respiratory pathogens [11]. Of note, the authors did not find any homology between SARS-CoV-2 and the human genome or proteome that would support a molecular mimicry mechanism. The lack of even a short, linear homology between the SARS-CoV-2 structure proteins and any axonal or myelin surface proteins reduces the likelihood that molecular mimicry with SARS-CoV-2 might be a putative mechanistic link of SARS-CoV-2 to GBS. On this issue, Keddie did not completely exclude a possible immunological similarity between SARS-CoV-2 and human proteome supporting a molecular mimicry mechanism. Actually, the lack of significant homologous protein between the human genome and SARS-CoV-2 genetic or linear protein structure in this study makes molecular mimicry as a cause less likely, but not excluding the possibility that immunogenic protein can be generated by post-translation modification sites or non-linear antibody epitopes theoretically [13]. Moreover, considering that SARS-CoV-2 can invade the nervous system via neural–mucosal interface in the olfactory mucosa in the nose and the gangliosides is abundant in olfactory nerves, the destruction of the olfactory epithelium and neuro-inflammation of the olfactory are considered to be the possible source of antigen expression [28]. While further research is required to demonstrate a causal relationship between SARS-CoV-2 and GBS.

To conclude, evidence establishing causal relationship between COVID-19 and GBS derive from two decisive aspects: (i) the need for rigorous case–control studies; (ii) the systematic detection of SARS-CoV-2 antibodies in patients with GBS including patients tested negative with PCR [28].

### Immunopathogenesis of GBS associated with COVID-19

GBS has been widely recognized as an immune-mediated disease with the participation of cellular and humoral immune responses, involving demyelination and axonal degeneration. However, the exact etiology and pathogenesis of GBS are not completely clear. As an immune-mediated inflammatory polyneuropathy, occurrence of GBS is related to prior infections caused by kinds of pathogens, such as viral or bacterial infections, particularly *C. jejuni*. Additionally, it has been reported that other antecedent non-infection events, including surgery, vaccines, immune checkpoint inhibitors (ICIs), pregnancy, and systemic lupus erythematosus, might also be triggers of GBS (Table 2) [29].

**Table 2:** Potential pathogenesis of GBS by infectious and non-infectious triggers

Triggers of GBS	Potential pathogenesis
Infectious events	
Bacteria	
* C. jejuni*	Molecular mimicry (GM1 epitopes on LOS from *C. jejuni*) and cross-reactivity between *C. jejuni* and peripheral nerve gangliosides
* M. pneumoniae*	Molecular mimicry between GM1 ganglioside and* M. pneumoniae*
* H. influenzae*	Molecular mimicry: presence of GM1 and GQ1b epitopes in *H. influenzae*
* Salmonella species*	Unclear
Viruses	
CMV	Molecular mimicry: presence of GM2 and GD2 epitopes on CMV, homology between moesin (a cytoskeletal component, key role on myelination) and a CMV phosphoprotein
HSV	Molecular mimicry: presence of GQ1b epitope on HSV-infected human fibroblasts
VZV	Unclear
EBV	Molecular mimicry? (anti-GQ1b antibodies); Vasculitis and neuritis caused by direct invasion
Hepatitis virus	Molecular mimicry? (anti-GM1 and GM2 antibodies); Vasculitis and neuritis caused by immune complex accumulation
Influenza virus	Molecular mimicry between glycoproteins of influenza viruses and glycolipids of peripheral nerves; Secondary infection
JEV	Unclear
HIV	Direct damage to nerves; Abnormal immunoregulation (formation of anti-sulfatide antibodies against myelin)
ZIKV	Direct damage to nerves (neurotropism); Molecular mimicry: peptide sharing between ZIKV proteins and human myelin protein-associated GBS
SARS-CoV-2	Unclear; Molecular mimicry? cytokine storm? abnormal activation of T or B cells? direct invasion? complement participation and mediation?
Non-infectious events	
Vaccines	Unclear; Molecular mimicry? (formation of anti-GM1antibodies induced by influenza vaccines)
Surgery	Immune dysfunction caused by surgery; Transient immunosuppression; Increased risk of secondary infection; Physical compression of the nerves; Activation of the endocrine stress systems
Exogenous gangliosides treatment	Immunogenicity of exogenous ganglioside (producing IgG anti-ganglioside antibodies)
ICIs	Imbalances in immunologic tolerance; Molecular mimicry? (shared GM2 and GD3 epitopes between melanoma and myelin sheath)
Pregnancy	Transient immunosuppression; Increased risk of opportunistic infections
SLE	Increased susceptibility to infections; Humoral immune response: autoantibodies against peripheral nerve myelin in GBS (a part of spectrum of autoantibodies observed in SLE); More potent response to neurogenic protein

*GBS* Guillain-Barré syndrome, *C. jejuni Campylobacter jejuni*, *LOS* lipooligosaccharide, *M. pneumoniae* Mycoplasma pneumoniae, *H. influenzae* Haemophilus influenzae, *CMV* Cytomegalovirus, *HSV* Herpes simplex virus, *VZV* Varicella-zoster virus, *EBV* Epstein-Barr virus, *JEV* Japanese encephalitis virus, *HIV* Human immunodeficiency virus, *ZIKV* Zika virus, *SARS-CoV-2* Severe acute respiratory syndrome coronavirus 2, *ICIs* immune checkpoint inhibitors; *SLE* Systemic lupus erythematosus.

**Fig. 1:**
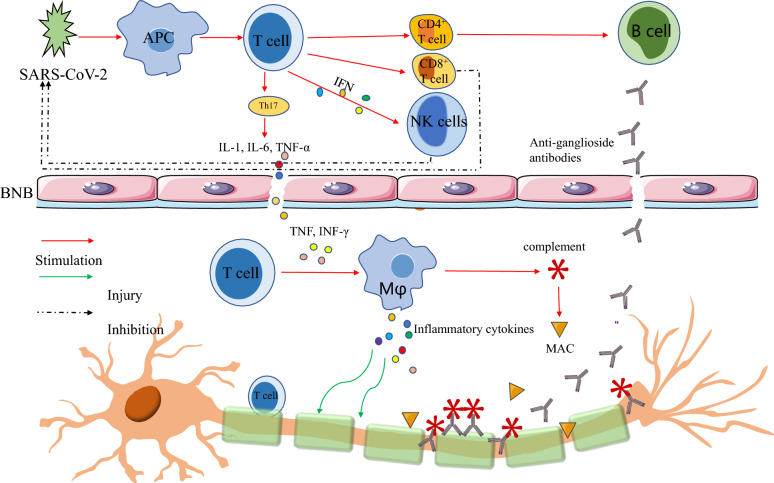
Possible pathogenesis and therapeutic strategy of GBS associated with SARS-CoV-2 infection. SARS-CoV-2 infection can induce immune-mediated peripheral nerve injury and ultimately lead to GBS likely because of the similar antigenic epitopes between pathogens and human peripheral nerve gangliosides (molecular mimicry and cross-reactivity) (anti-ganglioside antibodies have been detected in serum or CSF of GBS patients). When the S1 protein of the new coronavirus binds to ACE2R of human cells, it is recognized and captured by APC and presented to T cells. CD4 T cells stimulate B cells to produce anti-ganglioside antibodies and CD8 T cells directly kill virus-infected cells. Activated T cells secrete IFN, which stimulate NK cells to kill SARS-CoV-2. Moreover, activated T cells destroy the BNB by secreting IL-1, IL-6, and TNF-α to promote immune cells, complement, and antibodies crossing BNB to entry the CNS. In the peripheral nervous system, macrophages are activated by TNF and IFN-γ secreted by activated T cells. The activated macrophages release inflammatory cytokines to damage the myelin sheaths and axons of peripheral nerves. Besides, the autoantibodies can combine with the specific antigenic epitopes of myelin and axons to activate complement, inducing the formation of MAC, and macrophage migration and recruitment, ultimately leading to peripheral nerve injury. Besides, complement activation, MAC deposition, and infiltration of macrophages participate in the pathogenesis of GBS. In terms of therapy of GBS, IVIG plays a therapeutic role by neutralizing antibodies, blocking Fc receptors on macrophages, inhibiting complement activation and the formation of MAC, and regulating T and B cell functions. PE may work via scavenging autoantibodies, complement, MAC, and cytokines. Complement inhibitors, such as eculizumab, may be a potential therapy, and its therapeutic mechanism is associated with inhibition of complement activation and the formation of MAC. *SARS-CoV-2* acute respiratory syndrome coronavirus 2, *GBS* Guillain-Barré syndrome, *CSF* cerebrospinal fluid, *S1* Spike, *ACE2R* angiotensin converting enzyme 2 receptor, *APC* antigen-presenting cell, *TNF* tumor necrosis factor, *IL* interleukin, *TNF* tumor necrosis factor, *BNB* blood–nerve barrier, *CNS* central nervous system, *MAC* membrane attack complex, *Mφ* macrophages; *IVIG* Intravenous immunoglobulin, *PE* plasma exchange^+^^+^

*C. jejuni* is the most common pathogen that triggers GBS, which can generate antibodies through molecular mimicry mechanism and cross-reactivity, mostly resulting in immune attacks on the peripheral nerves and roots [2]. According to the reported cases of GBS after COVID-19 infection, anti-ganglioside antibodies (anti-GM1, GD1a, GD1b, GQ1b, and GT1b antibodies) have been detected in serum or CSF of patients [30]. The presence of antibodies implies that GBS caused by COVID-19 infection may be related to immune attack induced by molecular mimicry, with the cross-reactions between SARS-CoV-2 and human cell surface gangliosides (Fig. 1, SARS-CoV-2 infection can induce immune-mediated peripheral nerve injury and ultimately lead to GBS likely because cellular and humoral immune responses are activated by similar antigenic epitopes between pathogens and human peripheral nerve gangliosides) [31]. In addition, SARS-CoV-2 contained two immunologically related hexapeptides, belonging to the 41 human proteins associated with GBS, which is similar to the human heat shock proteins 90 (HSP90B and HSP90B2) and 60 (HSP60), respectively, supporting that molecular mimicry as a potential pathogenic mechanism of neuropathy after SARS-CoV-2 infection [32].

Due to elevated levels of the proinflammatory molecules, the increased cytokines and chemokines have been seen in COVID-19 patients, such as interleukin (IL)-1ß, IL-2, IL-6, IL-17, tumor necrosis factor (TNF-α), interferons (IFN-γ), and chemokines (CXCL8 and CXCL10), which play a pivotal role in both pathogenesis of GBS and the rapid progression of GBS associated with COVID-19 (Fig. 1). During the acute phase of GBS, the level of IL-17A, a prototypic IL-17 family member, was markedly elevated, which coordinates local tissue inflammation through upregulation of proinflammatory cytokines [33]. FTY720, a compound can ameliorate clinical severity of GBS via reducing IL-17A levels reported by a German group, which is mainly produced by Th17 cells. TNF-α, a transmembrane type II polypeptide precursor, has been identified as a key mediator in the pathogenesis of GBS. TNF-α is a Th1 cytokine with both proinflammatory and anti-inflammatory properties; however, TNF-α mainly played an inflammatory role in GBS and its level was related to clinical severity of disease [34]. With emerging evidence pointing to a role of IL-17 in the pathogenesis of GBS, TNF-α antagonists may lead to a rebound of Th17 cells in case series treated by anti-TNF-α, subsequently lead to an uncontrollable proinflammatory cascade [35]. Further studies are still needed to clarify the advantages and disadvantages of TNF-α antagonists in the treatment of GBS. IFN-γ is primarily produced by NK cells and NKT cells exerting its proinflammatory role by activating endothelial cells, macrophages, and T cells [32]. The serum level of IFN-γ was observed to elevate during the acute phase of GBS; nevertheless, it has been found to be protective in GBS. Most of all, shifting the application of a monoclonal antibody to IFN-γ into the beneficial one is an attracting therapeutic hint. Collectively, modulation of cytokine function can be identified as a potential therapeutic target for the management of both COVID-19 and GBS [27].

The pathologic features of GBS include inflammation, segmental demyelination, and axonal loss of peripheral nervous system (PNS). In the patients with GBS, macrophages expressed high levels MHC-I and MHC-II, indicating that macrophage-mediated proinflammatory immune mechanism exerted a crucial role in the generation lesions in the PNS of GBS.

In particular, it has been shown that antibodies may induce macrophages to bind antigen and enhance phagocytosis of macrophages by activating the complement-dependent manner or receptor-mediated manner [36]. During the inflammatory response, the infiltration and activation of immune cells contribute to organ injury in patients with COVID-19. By defining the genes involved in GBS and COVID-19, we delineated the protein–protein interaction networks of these two diseases, in which TH17 cell differentiation and cytokine response were found to play a common role in both of them. In conclusion, Li and others suggested aberrant Th17 cell differentiation as a possible mechanism by which COVID- 19 heightened the risk of GBS [37]. A decrease in circulating regulatory T cells (Tregs), accompanied by an elevation in circulating T helper (Th-17, Th-22, Th-1) cells, has been shown in patients with GBS. Polymorphisms of genes encoding components of the cytokines, including proinflammatory cytokines, such as TNF-α and IL-6, have been investigated for potential associations with susceptibility to GBS and disease progression [35].

In Brazil and India, SARS-CoV-2 has been detected in CSF of GBS patients after COVID-19 infection, indicating that the virus may directly invade the PNS to lead to demyelination and axonal damage [38, 39]. A variety of neurological manifestations caused by COVID-19 infection, including GBS, optic neuritis, meningitis, encephalitis, and acute transverse myelitis associated with SARS-CoV-2, provide evidences for the neuroinvasive potential of SARS-CoV-2. However, the neurological lesions may also be due to hypoxia, impaired immune response, and viral infection-mediated cerebrovascular injury. Moreover, most of the tested CSF samples were negative for SARS-CoV-2 PCR in such patients, and the time interval from the onset of symptoms attributed to SARS-CoV-2 and the GBS ranged from 2 to 3.5 weeks supporting an immune mechanism rather than direct invasion [36]. Only a few of studies have shown the presence or low detection of SARS-CoV-2 in the CSF, which could not serve as consistent evidence of direct central nervous system (CNS) invasion of SARS-CoV-2.

The complement family is an important component of our innate immune response to viruses, serving as a first line of defense against bacterial and playing a crucial role in promoting inflammatory processes triggering inflammatory cytokine storm. It has been hypothesized that targeted complement inhibition treatment is worthy of consideration for preventing systemic inflammatory response and endothelial injury of COVID-19 patients. From this point of view, eculizumab, a fully humanized monoclonal antibody against the complement protein C5, is of great benefit to patients with GBS theoretically [37]. A recent study on complement inhibitors have shown that eculizumab may improve outcomes in individuals with GBS at six months from onset. However, eculizumab alongside IVIG treatment is superior to the placebo but with higher the risk of infections. Therefore, shifting the complement inhibitors into the beneficial one is an attracting therapeutic hint. More studies should clarify whether eculizumab can become the usual therapeutic target of GBS [40]. An epidemiology study from the UK, however, has shown a decline in the incidence of GBS during the COVID-19 outbreak, possibly due to social isolation reducing the chance of bacterial and other viral infections, which indicated no link between GBS and COVID-19 infection [13].

Here, we discussed the possible mechanisms behind the relationship between SARS-CoV-2 and neurologic diseases, especially GBS, that seem promising for future studies. Future prospective studies with a larger sample size are needed to validate our discovery and elucidate the exact mechanism more clearly.

GBS cases increased in the research of Gigli (0.65 cases/100,000) and the incidence of GBS showed a 2.6-fold increase in another study by Filosto during the COVID-19 pandemic peak [6, 7]. However, the number of GBS patients decreased in 2020 compared with in 2016-2019 (1.65–1.88 cases/100,000) in a study of UK hospitals [11]. Considering all important factors, for example, different ages, races, and regions of patient, it is impossible to compare the number of GBS patients simply [41].

## GBS and COVID-19 vaccination

At this time, the available COVID-19 vaccines include messenger RNA (mRNA)-based vaccines, recombinant adenoviral vector vaccines, and inactivated whole-virus vaccines. To date, the Food and Drug Administration (FDA) of U.S.A. has granted emergency use authorization for four COVID-19 vaccines: two mRNA vaccines, that are BNT162b2 (Cominarty; Pfizer BioNTech) and mRNA-1273 (Spikevax; Moderna), and one replication-deficient adenovirus-based vaccine, that is, Ad26.COV2.S (Johnson & Johnson). An additional vaccine based on a radically innovative approach, that is, recombinant protein nanoparticles, NVX-CoV2373 (Novavax), adjuvanted contains the SARS-CoV-2 spike protein and Matrix-M adjuvant, is authorized for emergency use as a two-dose primary series to prevent COVID-19. Two other adenovirus-based vaccines, ChAdOx1 nCoV-19 vaccine (AstraZeneca/Oxford or Vaxzevria), and COVID-Vac/Sputnik V (Gamaleya Institute) have been approved conditional marketing authorization in Europe. For other vaccine multiple candidates, some of which already are available for use in different countries. Among them, BBIBP-CorV vaccine (Sinopharm, Beijing Bio-Institute of Biological Products Co. Ltd.), an inactivated SARS-CoV-2 isolate, has been listed for emergency use by the World Health Organization (WHO), potentially expediting its global roll out [42].

The mRNA vaccines introduce mRNA into cells, usually via a lipid nanoparticle (LNP). Inside the human body, mRNA enters the human cell and instructs the cells to identify the spike protein on the surface of SARS-COV-2, the virus that causes COVID-19. Our bodies then recognize the spike protein as an invader and produce antibodies against it. Later, if these antibodies encounter the actual virus, they are ready to recognize and kill the virus before it can cause diseases. In some patients, this immune response can trigger autoimmune processes that lead to the production of self-antibodies against the myelin, causing GBS via this pathway [43]. The adenovirus-based vaccine, which induces antibodies against SARS‐CoV‐2 spike glycoprotein, can mimic an actual infection and theoretically produce GBS. Simian adenovirus, the vector used in the adenovirus-based vaccine, may also be a trigger for GBS [44]. Probably, the mechanism for GBS development is a less specific immune cascade triggered by the adenovirus vector to generate host antibodies and cross-reactions with proteins on nerves [45].

The association between GBS and vaccination has been long debated since initial reports of increased incidence of GBS after the swine influenza vaccine during the USA/New Jersey 1976 vaccination campaign. The occurrence of GBS has been reported after the use of vaccines for other conditions, including polio, tetanus, rabies, hepatitis B, and meningococcal disease. Following the start of the worldwide mass vaccination campaign, real‐world case reports of GBS after COVID‐19 vaccine have emerged.

As of July 24, 2021, 130 reports of presumptive GBS were identified in Vaccine Adverse Event Reporting System (VAERS) following Ad26.COV2.S vaccination (median age: 56 years; interquartile range [IQR]: 45–62 years; 77 men [59.7%]). The median time to onset of GBS following vaccination was 13 days (IQR, 10–18 days), 105 cases (81.4%) beginning within 21 days and 123 (95.3%) within 42 days. The clinical symptoms in 121 reports (93.1%) were serious, including 1 death. The US Centers for Disease Control and Prevention reported that the overall estimated observed-to-expected rate ratio was 4.18, meaning an absolute increase of 6.36 cases per 100 000 person-years for the Ad26.COV2.S vaccine [46].

Data derived from the English National Immunoglobulin Database from January to October 2021, found a 2.04‐fold increased risk for GBS (95% confidence interval [CI]: 1.60–2.60) within 28 days after the AstraZeneca vaccine administration, but not after the Pfizer vaccine [47].

A study analyzing all cases of GBS reported to the Mexican Ministry of Health by recipients receiving the BNT162b2 mRNA COVID-19 vaccine between December 24, 2020, and March 19, 2021. Seven cases of GBS were detected among first-dose recipients, and no cases were reported after second-dose administration. Data showed that GBS was infrequent among recipients receiving the BNT162b2 [48].

A retrospective study of a nationwide registry of GBS among recipients receiving SARS-CoV-2 vaccines in Mexico also found the unadjusted GBS incidence for mRNA-based vaccines is similar to a previous report, including mRNA-1273 and BNT162b2 among Ad26.COV2.S recipients, when compared with other vaccines. In this cohort, patients with axonal variants, known to develop a more severe disease course with worse functional outcomes, accounted for 60.5% [49].

In a head-to-head comparison of Ad.26.COV2.S vs mRNA vaccines, the incidence rate of confirmed GBS in the 1 to 21 days after receiving the Ad.26.COV2.S (Janssen) vaccine was 32.4 per 100 000 person-years, which was significantly higher than the background rate of GBS, while the incidence rate of confirmed GBS in the 1 to 21 days after mRNA vaccines was 1.3 per 100 000 person-years, which did not differ from the background rate. These findings suggest an increased risk of GBS after Ad.26.COV2.S vaccination [50].

Klugar and others found that viral vector vaccines were the predominant vaccine type administered in early-onset post-COVID-19-vaccination GBS and GBS occurring after the 1st vaccination dose [51].

As of 30 May 2022, China had reported more than 3.38 billion doses of COVID-19 vaccines with238215 cumulative post-vaccination adverse events, and an overall reported incidence of adverse events was 70.45 per 1 million. A total of 290 cases of GBS were among the reported adverse events. Generally, the reporting rate of adverse reactions to China's new crown vaccines is lower than the report level of other routine vaccinations in the country in 2020.

A cohort study conducted from January 1, 2021 to June 30, 2021 at the Birmingham University Hospital, UK found a 2.6‐fold increase in number of admissions for GBS during the study period, compared to the same period in the previous 3 years. Patients presenting with GBS after AstraZeneca vaccine had more frequent facial and bulbar involvement than the historical cases, and more commonly they had the GBS variant with bifacial weakness and distal paresthesia [52].

As of April 22, 2021, around 1.5 million individuals in India had been vaccinated with COVID-19 vaccines. Over 80% of these individuals (1.2 million) received the ChAdOx1-S/nCoV-19 vaccine. In this population, there were seven cases of GBS that occurred within 2 weeks of the first dose of vaccination. All seven patients developed severe GBS. The frequency of GBS was 1.4- to tenfold higher than that expected in this period. In addition, a higher rate than expected rate of bilateral facial weakness was observed [53]. In a large multi‐institutional study in Taiwan involving 18,269 healthcare workers who received AstraZeneca vaccine, one single case of GBS variant (bilateral facial palsy with paresthesia) after the first vaccine dose was identified [54]. Pegat and others found a higher frequency of GBS occurring after adenovirus-vectored vaccines [55].

Patients with post-COVID-19 vaccination GBS, admitted to the six hospitals that cover the whole Liguria Region, Northwestern Italy, from February 1 to October 30, 2021, were included. The findings confirmed that most post-COVID-19 vaccination GBSs belong to the AIDP subtype, mainly characterized by sensory involvement, and occurred after the first vaccine dose, and that bilateral seventh cranial nerve involvement was not an uncommon manifestation [56].

Compared with classical GBS, patients with GBS related to COVID-19 vaccination showed unique clinical features, which include a more severe manifestation of the syndrome and more frequent bifacial diplegia. Hence, it is important for clinicians to have a high index of awareness to identify potential cases of bifacial weakness with paresthesias variant GBS following vaccination for SARS-CoV-2.

Considering the growing number of individuals vaccinated for COVID-19 worldwide, such reports of GBS will increase in the near future. Even so, the causal relationship between COVID-19 vaccines and GBS development should be considered with caution. Because of the heterogeneity of the sources of information, associations between COVID-19 vaccine and GBS must be confirmed in clinical epidemiological studies. In any case, the risk of GBS is not considered a legitimate reason to limit administration of currently available COVID-19 vaccines considering the efficacy of these vaccines in preventing COVID-19.

From the data that can be looked up, the incidence of GBS following SARS-CoV-2 infection fluctuated from 0.12 per 100,000 person-years to 9.44 per 100,000 person-years [6, 8], while after receiving the Ad.26.COV2.S (Janssen) vaccine was 32.4 per 100 000 person-years [50]. It seemed that people were more susceptible to be GBS when vaccinated, actually, the data about GBS related to vaccinations are incomplete [57]. Therefore, it is hard to compare the rate of GBS after SARS-COV-2 infection and after COVID-19 vaccination reasonably.

## Conclusions

In this review, we summarized recent developments in the understanding of the possible molecular mechanisms in SARS-CoV-2 infection causing GBS. At present, worldwide efforts for prevention of COVID-19 have focus on developing effective vaccines. Yet despite extensive efforts to develop vaccines against COVID-19, some vaccinated individuals still got infected even after the wide introduction of vaccine for COVID-19. Thus, there is a major need to understand better the molecular basis of SARS-CoV-2 infection in order to develop effective therapeutic strategies.

## Data Availability

Not applicable.

## Abbreviations

GBS: Guillain-Barré syndrome
CSF: Cerebrospinal fluid
*C. jejuni*: *Campylobacter jejuni*
LPSs: Lipopolysaccharides
AIDP: Acute inflammatory demyelinating polyneuropathy
AMAN: Acute motor axonal neuropathy
AMSAN: Acute motor and sensory axonal neuropathy
IVIG: Intravenous immunoglobulin
PE: Plasma exchange
SARS-CoV-2: Severe acute respiratory syndrome coronavirus 2
COVID-19: Coronavirus disease 2019
EDs: Emergency departments
RNA: Ribonucleic acid
UK: United Kingdom
U.S.A.: United States of America
IGOS: International Guillain-Barre´ syndrome Outcome Study
MFS: Miller Fisher syndrome
PCB: Pharyngeal-cervical-brachial
PCR: Polymerase chain reaction
ICIs: Immune checkpoint inhibitors
HSP: Human heat shock proteins
IL: Interleukin
TNF: Tumor necrosis factor
IFN: Interferons
CXCL: Chemokines
PNS: Peripheral nervous system
Tregs: Regulatory T cells
Th: T helper
CNS: Central nervous system
mRNA: Messenger ribonucleic acid
FDA: Food and Drug Administration
WHO: World Health Organization
LNP: Lipid nanoparticle
VAERS: Vaccine Adverse Event Reporting System
IQR: Interquartile range
CI: Confidence interval
